# Changes in the Amino Acid Composition of Bee-Collected Pollen During 15 Months of Storage in Fresh-Frozen and Dried Forms

**DOI:** 10.3390/foods15020207

**Published:** 2026-01-07

**Authors:** Aurita Bračiulienė, Rosita Stebuliauskaitė, Mindaugas Liaudanskas, Vaidotas Žvikas, Neringa Sutkevičienė, Sonata Trumbeckaitė

**Affiliations:** 1 Department of Pharmacognosy, Faculty of Pharmacy, Lithuanian University of Health Sciences, Sukilėlių av. 13, LT-50162 Kaunas, Lithuania; rosita.stebuliauskaite@lsmu.lt (R.S.); mindaugas.liaudanskas@lsmu.lt (M.L.); sonata.trumbeckaite@lsmu.lt (S.T.); 2 Institute of Pharmaceutical Technologies, Faculty of Pharmacy, Lithuanian University of Health Sciences, Sukilėlių av. 13, LT-50162 Kaunas, Lithuania; vaidotas.zvikas@lsmu.lt; 3 Animal Reproduction Laboratory, Faculty of Veterinary Medicine, Lithuanian University of Health Sciences, Tilžės Str. 18, LT-47181 Kaunas, Lithuania; neringa.sutkeviciene@lsmu.lt; 4 Laboratory of Biochemistry, Neuroscience Institute, Lithuanian University of Health Sciences, Eivenių Str. 4, LT-50162 Kaunas, Lithuania

**Keywords:** frozen and dried bee pollen, storage time, amino acids, essential and non-essential amino acids

## Abstract

Bee pollen (BP) is a nutritionally valuable natural product whose biological activity is strongly influenced by its amino acid profile. This study evaluated qualitative and quantitative changes in free amino acids in Lithuanian BP subjected to freezing (−20 °C and −80 °C) or low-temperature drying and stored for 15 months. Seventeen amino acids, including all nine essential amino acids, were identified using UHPLC-ESI-MS/MS, accounting for 47–48% of the total amino acid content (TAAC). Arginine, proline, and aspartic acid were the predominant free amino acids. Both frozen and dried samples showed a statistically significant decrease in TAAC after nine months of storage (*p* < 0.05), resulting in a 1.5–1.7-fold reduction after prolonged storage. Frozen storage at −20 °C and −80 °C better preserved free amino acids, particularly alanine, glutamic acid, and proline, whereas dried BP stored at room temperature exhibited accelerated degradation. Sulfur-containing amino acids, especially cysteine and methionine, were highly unstable under all storage conditions. These results provide practical guidance for selecting storage strategies that minimize amino acid losses and help maintain the nutritional quality of bee pollen during long-term storage.

## 1. Introduction

Bee pollen (BP) is a conglomerate of plant pollen grains collected by worker bees and agglutinated with nectar, honey, and glandular secretions [1]. It is considered a valuable natural bioproduct containing more than 200 biologically active compounds with potential therapeutic effects [2,3,4]. The beekeeping sector produces a wide range of food products and nutraceuticals [5,6]. However, compared to honey, royal jelly, and propolis, bee-collected pollen has received relatively limited scientific attention to date [7,8].

Research has shown that BP exhibits antioxidant, anti-inflammatory, hepatoprotective, anti-atherosclerotic and other biological properties, and provides protective effects on the digestive and nervous systems [9,10,11,12,13,14]. These biological effects are closely related to its complex chemical composition, which includes both primary (e.g., proteins: 2–60%, carbohydrates: 13–55%, lipids: 1–20%) and secondary (e.g., phenolic compounds: 3–5%) metabolites [1,4,15,16,17,18]. The protein content of BP is often regarded as an indicator of its nutritional value [17,19,20]. However, several studies suggest that amino acid composition provides a more accurate measure of nutritional quality, as the absence or insufficiency of essential amino acids can significantly reduce their biological value [21].

Amino acids are vital compounds characterized by unique chemical properties. They serve as the monomeric units of proteins and play fundamental roles in numerous biological processes. While their primary function is protein synthesis, amino acids also participate in the biosynthesis of hormones, enzymes, neurotransmitters, and other biologically active molecules. Additionally, they contribute to nitrogen metabolism and support the structural and functional integrity of cells [22,23,24]. Amino acid requirements vary depending on species, developmental stage, physiological condition, intestinal microbiota composition, environmental factors, and pathological states [24,25,26]. Numerous studies have demonstrated beneficial effects of amino acids in the prevention and treatment of health conditions including infertility, gastrointestinal disorders, neurological dysfunction, cardiovascular diseases, atherosclerosis, and diabetes mellitus [27,28,29].

According to the Food and Agriculture Organization (FAO), BP may contain more than 16 distinct amino acids and numerous non-protein amino acids, further emphasizing its nutritional significance [4,30]. Amino acids are generally categorized as nutritionally essential or non-essential [24]. Essential amino acids—such as leucine, isoleucine, methionine, phenylalanine, arginine, histidine, tryptophan, valine, threonine, and lysine—cannot be synthesized by the human body and therefore must be obtained through diet. In contrast, non-essential amino acids, including alanine, asparagine, cysteine, glutamic acid, aspartic acid, glycine, proline, serine, and tyrosine, can be synthesized endogenously by both plants and humans [31]. Consequently, BP as a functional food ingredient represents a valuable dietary source of essential amino acids.

The chemical composition of BP, including the qualitative and quantitative profile of amino acids, is influenced by factors such as botanical and geographical origin [32,33], as well as storage and processing conditions [34]. Freshly harvested pollen collected by honeybees contains approximately 15% to 30% (*w*/*w*) of moisture. As a result, immediate processing is necessary to enhance physicochemical stability and prevent microbial growth [35,36]. Traditional drying techniques, which are often poorly standardized, typically involve hot air drying that exposes the pollen to elevated temperatures. This method may negatively affect the content of antioxidants and lipids [7,37]. Hot air drying is also considered detrimental to proteins, as elevated temperatures can induce thermal stress and lead to conformational instability [38]. To address these limitations, recent studies have explored alternative drying methods that operate at lower temperatures, such as freeze-drying, to reduce moisture content in bee-collected pollen [39]. These techniques have proven effective in minimizing the degradation of sensitive compounds while preserving the functional properties of BP [7,40,41,42].

Previous studies on BP have primarily focused on the effects of botanical origin [43], drying techniques, or short-term storage [23,44], and on general nutritional composition [45,46,47]. A comparative overview of these studies, including storage duration, temperature range, and amino acid–related parameters evaluated, is summarized in Supplementary Table S1. However, comprehensive quantitative data on long-term changes in free amino acid profiles during storage—particularly under frozen conditions at different temperatures—remain scarce. Moreover, degradation kinetics of individual amino acids in BP have rarely been investigated, limiting mechanistic understanding of amino acid stability and quality loss during extended storage. In Lithuania, dried BP has traditionally been consumed, whereas in several other countries the use of fresh-frozen pollen is becoming increasingly common. Despite these differing practices, systematic comparisons of amino acid stability under low-temperature drying and freezing at −20 °C and −80 °C over prolonged storage periods are lacking. In particular, little is known about how essential and non-essential amino acids respond to different storage temperatures over time and whether ultra-low temperatures consistently provide superior preservation.

Therefore, the aim of the present study was to investigate qualitative and quantitative changes in free amino acids in Lithuanian BP preserved by low-temperature drying or immediate freezing at −20 °C and −80 °C during a 15-month storage period. Amino acid composition was monitored at three-month intervals using UHPLC-ESI-MS/MS to capture dynamic changes over time. In addition, first-order degradation kinetics were applied to individual amino acids to quantitatively assess degradation rate constants and model accuracy. This study provides novel insights into the long-term stability of free amino acids in BP and clarifies the role of storage temperature and preparation method in preserving its nutritional quality.

## 2. Materials and Methods

### 2.1. Collection of Bee Pollen

Bee pollen was collected during April–May 2024 from a single apiary located in Talkoniai, Pasvalys District, Lithuania (coordinates: 55.9598° N, 24.3422° E), with all samples originating from the same harvest period. Pollen loads were harvested using pollen traps under favorable weather conditions during peak flowering periods. The collected fresh bee pollen was cleaned and allocated for storage according to the experimental conditions described below. Freshly collected samples were either immediately frozen or dried and subsequently analyzed throughout the study.

The botanical composition of the BP was determined in our previous study [48]. Melissopalynological analysis revealed that *Salix* spp. and *Brassica napus* L. were the dominant pollen types in all samples, accounting for 34.3% and 36.8% of the total pollen content, respectively. *Acer platanoides* L., *Malus domestica* Borkh., and *Taraxacum officinale* L. were identified as minor pollen contributors, representing 12.8%, 9.0%, and 5.9% of the pollen spectrum, respectively.

### 2.2. Bee Pollen Preparation

#### 2.2.1. Preparation of Dried Bee Pollen Samples

Freshly collected BP samples were dried in a controlled drying chamber, maintaining a temperature of +28 °C for the first 24 h and +35 °C for the subsequent 24 h. The dried material was stored in airtight containers in a dry, dark, and well-ventilated room at ambient temperature (+19–21 °C) throughout the study period.

#### 2.2.2. Preparation of Frozen Bee Pollen Samples

Immediately after collection, BP samples were frozen at −20 °C and −80 °C using laboratory-grade freezers. The samples were stored in sealed containers at constant temperatures (−20 °C and −80 °C, respectively) for the duration of the experiment. The frozen material was processed immediately after extraction. Qualitative and quantitative variations in the composition of the BP were assessed every three months. All results were recalculated on a dry weight basis.

To ensure sample independence and to avoid repeated freeze–thaw cycles, BP samples were pre-aliquoted into multiple sealed containers immediately after preparation (freezing at −20 °C and −80 °C or low-temperature drying). Each container represented an independent sample and was opened and analyzed only once at the corresponding storage time point (0, 3, 6, 9, 12, and 15 months). At each time point, three independent aliquots were analyzed (*n* = 3). Samples collected at different time points were therefore treated as independent observations.

### 2.3. Chemicals

All solvents, reagents, and standards used were of analytical grade. The solvents used were as follows: purified water (Millipore^®^, Bedford, MA, USA), ethanol 96.0% (*v*/*v*) (AB Vilnius degtinė, Vilnius, Lithuania). The standards of amino acids used were as follows: L-alanine, L-aspartic acid, L-arginine, L-phenylalanine, L-proline, L-leucine, L-glycine, L-tryptophan, L-valine, L-asparagine, L-isoleucine, L-methionine, L-cysteine, L-threonine, L-glutamic acid, L-serine, L-lysine, L-glutamine, L-histidine, L-tyrosine (Sigma-Aldrich^®^, Steinheim, Germany). For UHPLC-ESI-MS/MS analysis, ammonium formate, formic acid (≥98%), and LC−MS grade acetonitrile were used as high-purity reagents and mobile-phase components (Sigma-Aldrich^®^, Steinheim, Germany).

### 2.4. Preparation of Ethanol Extract from Bee Pollen

The procedure for preparing ethanol extracts of BP was adapted from the method described by Kahraman et al. [49]. Exactly 1.0 g of homogenized BP—consisting of crushed, dried, and frozen samples (stored at −20 °C and −80 °C)—was weighed. The measured sample was transferred into identical, dark glass vials and mixed with 10 mL of 70.0% (*v*/*v*) ethanol. Three replicates of each BP type were prepared (*n* = 9). The samples were subjected to sonication in an ultrasonic bath for 50 min. Following this, the mixture was vacuum-filtered using a system connected to a vacuum pump, separating the solid residue from the liquid extract. The final volume of the liquid phase was adjusted to exactly 10 mL. The remaining solid residue on the filter was rinsed with a 70.0% (*v*/*v*) ethanol solution to ensure complete extraction. The resulting ethanol extracts were transferred into sealed dark glass bottles, labeled accordingly, and stored at room temperature in the absence of light until further analysis. These extracts were subsequently used for the identification and quantification of amino acids.

### 2.5. UHPLC-ESI-MS/MS Method for the Qualitative and Quantitative Analysis of Free Amino Acids

Amino acid UHPLC-ESI-MS/MS analysis were performed using a method adapted from Taujenis L. [50]. Amino acid analysis of BP extracts was carried out using an Acquity H-Class UPLC system (Waters, Milford, MA, USA) coupled to a Xevo TQD mass spectrometer (Waters, Milford, MA, USA). A 1.0 µL aliquot of extract was injected onto a BEH Amide column (150 mm × 2.1 mm, 1.7 µm; Waters), with the column temperature maintained at 25 °C. The mobile phase consisted of 10 mmol ammonium formate with 0.125% formic acid (eluent A) and acetonitrile (eluent B), delivered at a flow rate of 0.6 mL/min. Gradient elution was applied as follows: 0–1 min, 95% B; 1–3.9 min, 70% B; 3.9–5.1 min, 30% B; 5.1–6.4 min, 70% A for column flushing; and at 6.5 min, the gradient was returned to the initial conditions for a total runtime of 10 min. The mass spectrometer operated in positive electrospray ionization mode with a capillary voltage of +3.5 kV, cone voltage of 30 V, desolvation gas flow of 800 L/h at 400 °C, and source temperature of 120 °C. Amino acids in BP extracts were identified by comparing retention times and MS/MS spectra with those of analytical-grade standards, and quantification was performed using linear regression models from the standard dilution method. Mass spectrometer parameters for amino acid analysis are presented in Table 1.

### 2.6. Application of the First-Order Degradation Model

The degradation of free amino acids was modeled using the first-order kinetic equation: *C_t_* = *C*_0_ × *e*^−*kt*^, where

*C_t_* is the amount of amino acids at time *t*,

*C*_0_ is the initial content,

*k* is the degradation rate constant (month^−1^),

*t* is storage time in months.

For each amino acid, the estimated degradation rate constant *k* (month^−1^), and the determination coefficient (R^2^), which indicates the accuracy of the model, were calculated. A determination coefficient (R^2^) value approaching 1 indicates an excellent fit between the model and the observed data. This model provides a predictive and mechanistic understanding of compound stability and supports more precise interpretation of storage-related quality loss [51].

The calculated half-lives (*t*_1/2_) provide a quantitative measure of amino acid stability. Within the framework of the first-order kinetic model, *t*_1/2_ indicates the time required for the concentration of an individual amino acid to decline to 50% of its initial level at a given storage temperature, thereby allowing direct comparison of degradation rates among amino acids and storage conditions. For first-order kinetics, the half-life is calculated using the following equation: *t*_1/2_ = ln(2)/*k*, where

*t*_1/2_ is the half-life (months),

*k* is the degradation rate constant (month^−1^).

Longer half-lives indicate greater stability of amino acids during storage, whereas shorter half-lives reflect higher susceptibility to degradation.

During the study, Q_10_ coefficients for amino acids were calculated based on the formula: Q_10_ = (*k*_2_/*k*_1_) ^ (10/(T_2_ − T_1_)), where

*k*_1_—the degradation constant at the lower temperature,

*k*_2_—the degradation constant at the higher temperature.

The Q_10_ coefficient describes how the rate of a chemical or biochemical reaction changes when the temperature is altered by 10 °C.

### 2.7. Statistical Analysis

The UHPLC-ESI-MS/MS data were processed using Microsoft Office Excel 2015 (Microsoft Corp., Redmond, WA, USA) and SPSS Statistics 25 (IBM Corp., Armonk, NY, USA). All experiments were conducted in triplicate (*n* = 3), and the results are presented as the arithmetic mean ± standard deviation (SD). Samples analyzed at different storage time points corresponded to independently stored aliquots prepared at the beginning of the experiment and maintained under identical conditions until analysis, thereby avoiding repeated freeze–thaw cycles. Consequently, measurements at different time points were treated as independent samples in the statistical analysis. Statistical differences among groups were evaluated using one-way analysis of variance (ANOVA). The homogeneity of variances was assessed using Levene’s test, and when the assumption of equal variances was met, Tukey’s post hoc test was applied to identify pairwise differences. A significance level of *p* < 0.05 was used for all statistical tests. In addition, principal component analysis (PCA) was performed, considering only components with eigenvalues greater than one, to identify the main factors contributing to variation among the samples.

## 3. Results

### 3.1. Qualitative and Quantitative Profiling of Free Amino Acids by UHPLC-ESI-MS/MS

Using ultra-high performance liquid chromatography with electrospray ionisation mass spectrometry (UHPLC-ESI-MS/MS), a total of 17 free amino acids were identified in the BP extracts, including 9 essential (arginine (Arg), histidine (His), isoleucine (Ile), leucine (Leu), lysine (Lys), methionine (Met), phenylalanine (Phe), threonine (Thr), and valine (Val) and 8 non-essential amino acids (alanine (Ala), aspartic acid (Asp), cysteine (Cys), glutamic acid (Glu), glycine (Gly), proline (Pro), serine (Ser), and tyrosine (Tyr).

**Figure 1:**
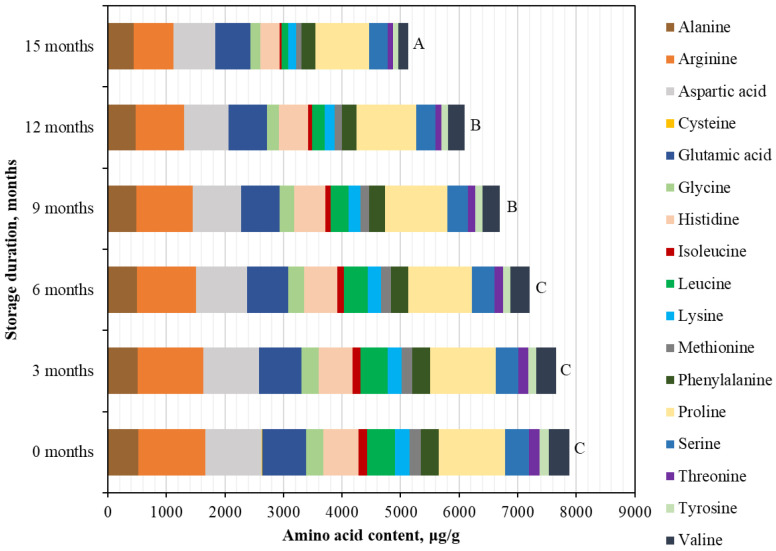
Quantitative changes in the profile of free amino acids in BP during 15 months of storage at −20 °C. Different letters indicate statistically significant differences in the total amount of identified free amino acids at different storage periods (*p* < 0.05).

Qualitative and quantitative analysis revealed that three amino acids—arginine, proline, and aspartic acid—predominated in BP samples stored at −20 °C (Figure 1). The highest concentrations were observed for Arg (1145.01 ± 46.45 μg/g, accounting for 14.5% of the total amino acid content (TAAC)), Pro (1133.89 ± 3.73 μg/g, accounting for 14.4% of the TAAC), and Asp (965.79 ± 25.10 μg/g, accounting for 12.3% of the TAAC) (Figure 1). In contrast, the lowest concentration was found for Cys (14.18 ± 1.51 μg/g), representing only 0.2% of the TAAC (Figure 1). After 15 months of storage at −20 °C, the content of individual free amino acids in BP samples decreased to varying degrees (in ascending order of reduction): Ala (−15.1%) > Glu (−18.5%) > Pro (−19.2%) > Ser (−21.4%) > Phe (−22.5%) > Asp (−26.3%) > Tyr (−37.8%) > Arg (−40.6%) > Gly (−41.9%) > Lys (−46.3%) > His (−46.5%) > Val (−50.2%) > Thr (−53.1%) > Met (−53.2%) > Ile (−72.9%) > Leu (−76.4%) > Cys (−99.9%) (Figure 1). For most amino acids, the decline during storage was gradual. However, cysteine exhibited a marked 1.4- to 2.9-fold decrease within the 3–6-month period, and after 15 months, its content was reduced to undetectable levels. Isoleucine and leucine showed a significant decrease after 6 months of storage, while methionine and valine demonstrated a pronounced 1.3- to 1.5-fold reduction between 12 and 15 months.

At the beginning of the storage period, the total content of identified free amino acids in BP samples stored at −20 °C was 7879.17 ± 393.96 µg/g, with no statistically significant differences observed after 3 and 6 months of storage (Figure 1). A statistically significant decrease (1.2-fold) in the total free amino acid content was observed only after 9 months of storage at −20 °C (*p* < 0.05). After 15 months, the total free amino acid content in the samples stored at −20 °C had decreased by a statistically significant 1.5-fold, reaching 5135.28 ± 256.76 µg/g (*p* < 0.05).

**Figure 2:**
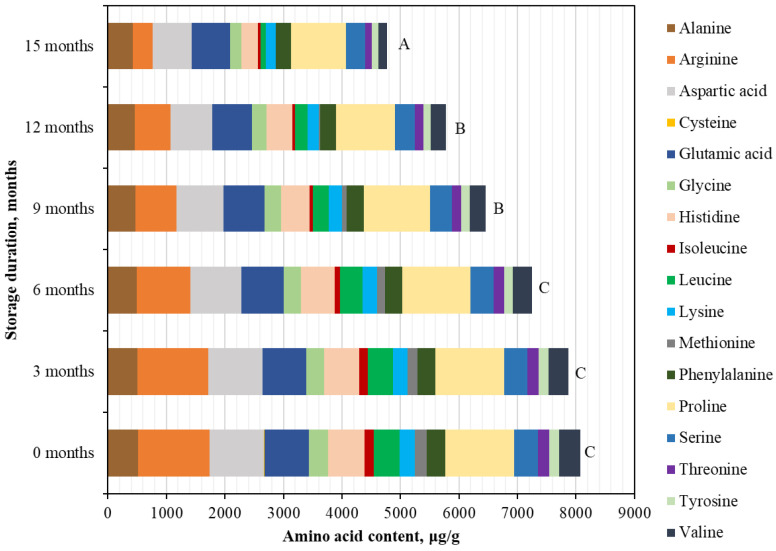
Quantitative changes in free amino acid composition in BP during 15 months of storage at −80 °C. Different letters indicate statistically significant differences in the total amount of identified free amino acids at different storage periods (*p* < 0.05).

Similarly, BP samples stored at −80 °C, Arg, Pro, and Asp were among the most abundant free amino acids, indicating a consistent dominance of these compounds under freezing conditions. At the beginning of the study, the highest concentrations were determined for Arg (1216.61 ± 11.77 μg/g, accounting for 15.1% of the TAAC), Pro (1176.03 ± 7.33 μg/g, accounting for 14.6% of the TAAC), and Asp (930.38 ± 46.52 μg/g, accounting for 11.5% of the TAAC). In contrast, the lowest concentration was found for Cys (13.65 ± 0.84 μg/g), representing only 0.2% of the TAAC (Figure 2). After 15 months of storage at −80 °C, changes in individual free amino acid levels in BP samples were observed in varying degrees (in ascending order of reduction): Glu (−14.2%) > Ala (−17.4%) > Phe (−19.4%) > Pro (−19.7%) > Ser (−21.4%) > Asp (−27.7%) > Lys (−34.9%) > Tyr (−35.9%) > Gly (−40.6%) > Thr (−41.5%) > His (−54.1%) > Val (−57.3%) > Arg (−72.3%) > Ile (−76.7%) > Leu (−77.5%) > Cys (−99.86%) > Met (−100%) (Figure 2). Methionine and cysteine were not detected after 15 months of storage, indicating complete degradation (−100%). Overall, the decline in free amino acid content was gradual across most amino acids. Exceptions included isoleucine, which exhibited a marked 1.6-fold decrease as early as 6 months. Leucine exhibited a consistent and statistically significant decrease at each three-month interval, whereas arginine and valine showed a pronounced reduction (1.7–1.9-fold) during the 12–15-month storage period.

At the beginning of the study, the total identified concentration of free amino acids in BP samples stored at −80 °C was 8073.97 ± 403.70 µg/g, showing no statistically significant difference compared with the content determined after 3 and 6 months of storage. A statistically significant 1.3-fold decrease in the total free amino acid content was observed after 9 months (*p* < 0.05). By the end of the study, following 15 months of storage at −80 °C, the total content of free amino acids in the BP samples had decreased 1.7-fold, reaching 4772.67 ± 238.63 µg/g (*p* < 0.05).

**Figure 3:**
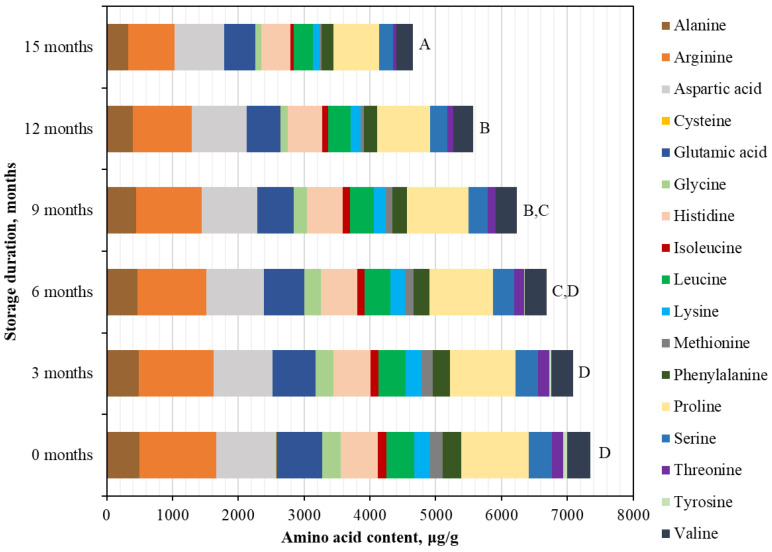
Quantitative changes in free amino acid composition in dried BP samples over a 15-month period. Different letters indicate statistically significant differences in the total amount of identified free amino acids at different storage periods (*p* < 0.05).

Similarly to the frozen BP samples, three major free amino acids—Arg, Asp, and Pro—predominated in the dried BP samples. At the beginning of the study, the concentration of Arg in BP samples was 1164.52 ± 58.23 µg/g, accounting for 15.8% of TAAC. The content of Pro was 1026.65 ± 51.33 µg/g, representing 14.0% of the TAAC, while Asp was present at 903.76 ± 45.20 µg/g, corresponding to 12.3% of the TAAC (Figure 3). After 15 months of storage, the dried BP samples exhibited the following changes in free amino acid content (in order of increasing decrease): Asp (−17.1%) > His (−21.2%) > Val (−28.8%) > Leu (−30.3%) > Glu (−31.2%) > Pro (−32.3%) > Phe (−34.3%) > Ala (−35.2%) > Arg (−39.0%) > Ser (−40.9%) > Lys (−57.5%) > Ile (−63.7%) > Gly (−70.1%) > Thr (−71.1%) > Met (−87.8%) > Tyr (−100%) > Cys (−100%) (Figure 3). Tyrosine was no longer detected after 9 months, whereas cysteine was absent already after 6 months of storage. The decrease in free amino acid levels was generally gradual across all amino acids, except for glycine, methionine, and threonine, which showed a 1.6–2.1-fold reduction between 9 and 12 months, and for isoleucine and lysine, which exhibited a 1.5–1.9-fold decrease between 12 and 15 months.

At the beginning of the study, the total content of free amino acids in the dried BP samples was 7352.02 ± 367.60 µg/g and did not change significantly during the first 6 months of storage. However, after 9 months, a statistically significant 1.1-fold decrease was observed (*p* < 0.05). A similar trend was noted after prolonged storage: following 15 months, the total free amino acid content in the dried BP samples, as well as in the frozen ones, decreased significantly by 1.6-fold, reaching 4648.33 ± 232.42 µg/g (*p* < 0.05).

**Figure 4:**
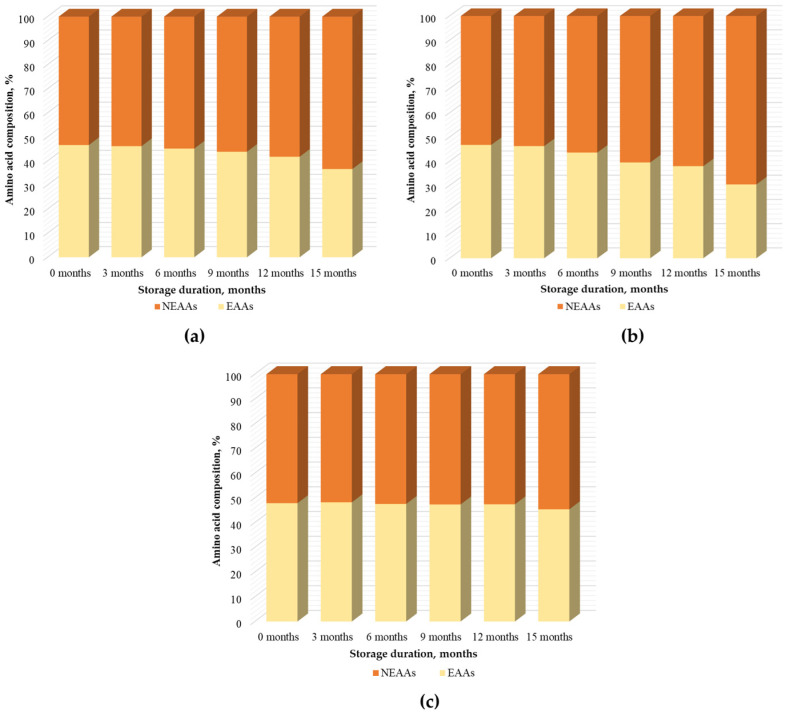
The percentage distribution of essential (EAAs) and nonessential amino acids (NEAAs) in BP samples is presented as follows: (**a**) percentage distribution of EAAs and NEAAs in BP samples stored at −20 °C; (**b**) percentage distribution of EAAs and NEAAs in BP samples stored at −80 °C; (**c**) percentage distribution of EAAs and NEAAs in dried BP samples.

Among the 17 identified and quantitatively determined free amino acids in the BP samples, 9 were classified as essential amino acids—Arg (arginine), His (histidine), Ile (isoleucine), Leu (leucine), Lys (lysine), Met (methionine), Phe (phenylalanine), Thr (threonine), and Val (valine)—which cannot be synthesized by the human body and therefore must be obtained from the diet. In the BP samples, the identified free essential amino acids (EAAs) accounted for 46.6% to 47.9% of the total amino acid content. In frozen BP samples, the EAA content gradually decreased during storage. After three months, the EAA content in frozen BP samples had decreased by only 0.5%. A smaller decline was observed in samples stored at −20 °C, where the EAA content decreased by 1.5%, 2.8%, 4.9%, and 10.0% after 6, 9, 12, and 15 months of storage, respectively. In contrast, a more pronounced reduction occurred in samples stored at −80 °C, with decreases of 3.1%, 7.2%, 8.7%, and 16.3% over the same periods. In dried BP samples, the EAA content remained relatively stable throughout the entire storage period, with variations ranging from 0.3% to 2.5%. After 15 months of storage, the EAA content in the dried BP samples decreased by only 2.5% (Figure 4).

**Figure 5:**
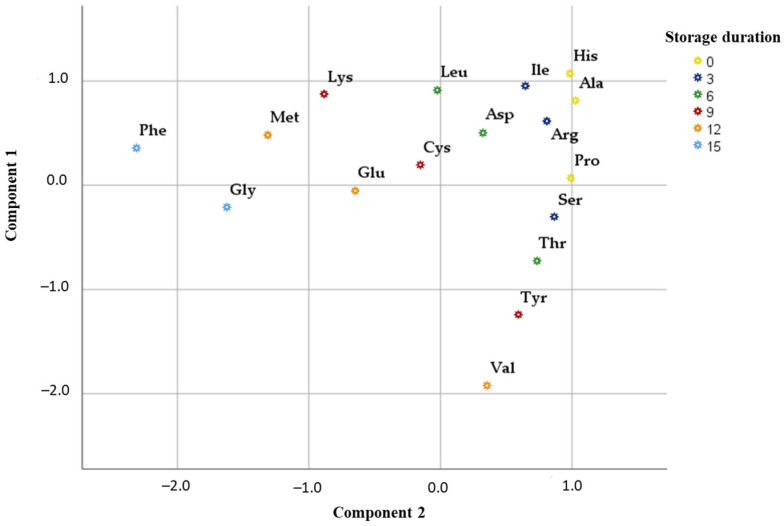
Principal component analysis plot that showed the distribution of amino acids according to storage duration in BP samples.

In this study, principal component analysis (PCA) was performed to determine the relationships between changes in free amino acid concentrations during different storage periods. Two main components were used for the analysis, as they explain 91.65% of the total variability in the research data. The analysis revealed that there was a very strong positive correlation between Glu (0.968), Phe (0.956), Pro (0.933), Ser (0.943), and Tyr (0.924) with the first component describing 46.76% of the total data dispersion. These amino acids exhibit a strong interrelationship and reflect specific biochemical mechanisms characteristic of the early phases of the storage process, which are marked by initial protein denaturation, hydrolysis, and oxidation reactions. We found a very strong positive correlation between Arg (0.955), Asp (0.940), His (0.926), Ile (0.915), Leu (0.961), and Val (0.945) with the second component describing 44.89% of the total data dispersion (Figure 5). The high loadings of these compounds indicate their greater significance during the later stages of the storage process, when amino acid deamination, transamination, and other secondary degradation processes intensify, reflecting advanced biochemical transformations.

The effect of storage duration is clearly reflected in the distribution of samples along the principal components. Initial samples (0 months, yellow dots) cluster near Pro and His, indicating the abundance of these amino acids in fresh samples. As storage time increases (9–15 months), the samples gradually shift toward the direction of Component 2, i.e., toward Val, Tyr, Arg, and Asp. This trend suggests that prolonged storage induces significant biochemical alterations associated with amino acid transformations and the formation of new compounds.

### 3.2. Application of the First-Order Degradation Model for the Evaluation of Amino Acid Stability During Storage

The kinetic modeling results strongly support the application of a first-order degradation model to describe the stability of free amino acids in bee pollen (BP) samples. The application of the first-order kinetic model enabled a quantitative assessment of the stability of individual amino acids during storage using degradation rate constants (k), coefficients of determination (R^2^), and half-lives (t_1/2_). Overall, the model provided both mechanistic insight and robust quantitative parameters for evaluating amino acid stability under different storage conditions: (1) The high coefficients of determination (R^2^ = 0.68–1.00) indicate that the first-order kinetic model accurately describes the degradation processes of free amino acids in both frozen (−20 °C and −80 °C) and dried BP samples; (2) Cysteine was found to degrade the fastest under all tested conditions, with degradation rate constants ranging from *k* = 0.4488 month^−1^ (R^2^ = 0.9444) to *k* = 0.9024 month^−1^ (R^2^ = 1.0000), corresponding to half-life values (t_1/2_) between 0.77 and 1.54 months; (3) The results indicate that storage at –80 °C provides the greatest amino acid stability, whereas drying promotes the most extensive degradation, particularly for oxidation-prone compounds such as cysteine, methionine, and tyrosine.

**Figure 6:**
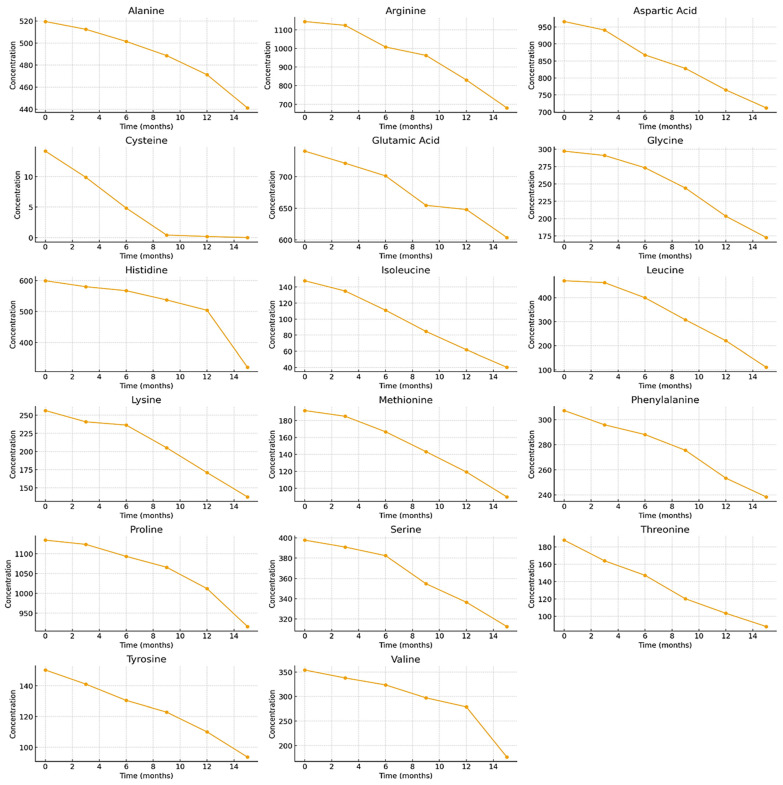
Time-dependent degradation curves of amino acids in BP samples stored at −20 °C.

The results of the study revealed pronounced differences in amino acid stability. Cysteine exhibited the highest degradation rate (*k* = 0.4488 month^−1^) and the shortest half-life (t_1/2_ = 1.54 months). In BP samples, cysteine almost completely disappeared after 15 months of storage at −20 °C. Isoleucine and leucine also degraded relatively rapidly (t_1/2_ ≈ 7.5–8.0 months), indicating moderate stability. In contrast, amino acids such as alanine, proline, glutamic acid, and serine exhibited the lowest degradation rate constants (*k* ≈ 0.010–0.016 month^−1^) and the longest half-lives (t_1/2_ > 40 months), indicating high stability under the investigated storage conditions. Alanine was among the most stable amino acids (t_1/2_ = 66.36 months), despite its relatively high initial concentration (Table 2, Figure 6).

The high coefficients of determination (R^2^ = 0.6817–0.9857) indicate that the first-order kinetic model accurately describes the degradation processes of amino acids in BP samples stored at −20 °C. In this study, threonine (R^2^ = 0.9941) and aspartic acid (R^2^ = 0.9857) exhibited the best agreement with the first-order degradation model, indicating that their degradation can be reliably predicted over time (Table 2, Figure 6).

**Figure 7:**
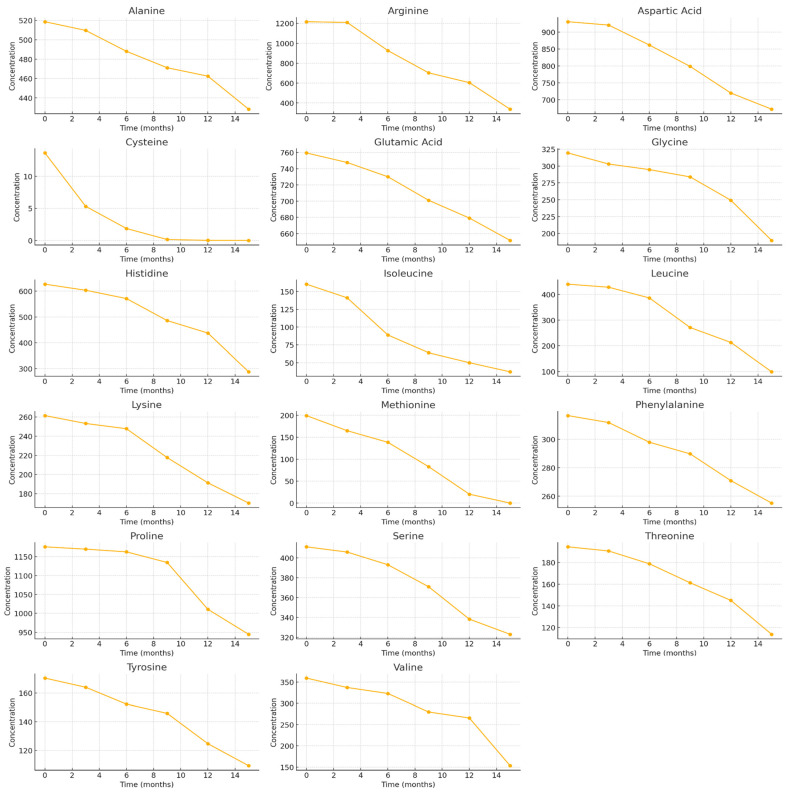
Time-dependent degradation curves of amino acids in BP samples stored at −80 °C.

Analysis of BP samples stored at −80 °C revealed differences in the stability of individual amino acids over a 15-month storage period. The highest degradation rate was observed for cysteine (*k* = 0.6025 month^−1^), with a half-life of only 1.15 months, confirming the extremely low stability of this amino acid. Similarly high degradation rates were determined for methionine (*k* = 0.1756 month^−1^; t_1/2_ = 3.95 months). In contrast, alanine, glutamic acid, phenylalanine, proline, and serine exhibited the lowest degradation rate constants (*k* ≈ 0.010–0.017 month^−1^) and the longest half-lives (t_1/2_ > 40 months), indicating their high stability under −80 °C storage conditions. Glutamic acid was among the most stable amino acids (t_1/2_ = 66.51 months), while alanine also demonstrated high stability (t_1/2_ = 56.86 months) (Table 3, Figure 7).

The high coefficients of determination (R^2^ = 0.7765–0.9864) confirm that the first-order kinetic model adequately describes the degradation behavior of amino acids under these conditions (Table 3, Figure 7).

**Figure 8:**
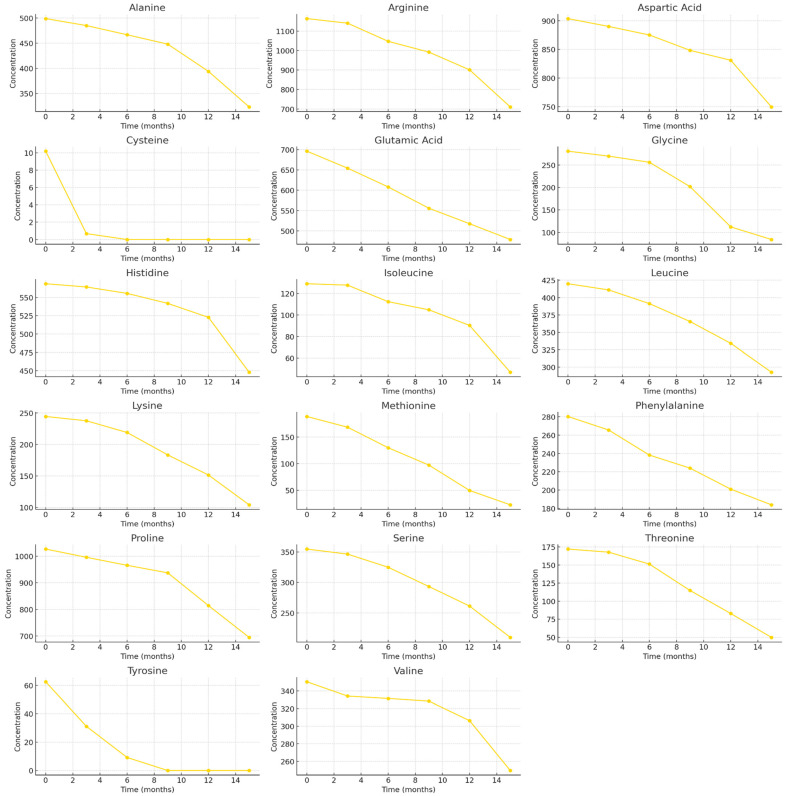
Time-dependent degradation curves of amino acids in dried BP samples.

Analysis of dried BP samples revealed that the stability of amino acids over a 15-month storage period varied depending on their molecular structure. The highest degradation rate was observed for cysteine (*k* = 0.9024 month^−1^), which exhibited a very short half-life (t_1/2_ = 0.77 months). The coefficient of determination (R^2^ = 1.0000) indicates an excellent fit of the kinetic model to the experimental data. These results confirm that cysteine is the most unstable amino acid under drying and room-temperature storage conditions. Similarly high degradation rates were observed for tyrosine (*k* = 0.3189 month^−1^; t_1/2_ = 2.17 months) and methionine (*k* = 0.1380 month^−1^; t_1/2_ = 5.02 months) (Table 4, Figure 8).

In contrast, amino acids such as aspartic acid, histidine, valine, leucine, proline, glutamic acid, and alanine exhibited lower degradation rate constants (*k* ≈ 0.011–0.027 month^−1^) and longer half-lives (t_1/2_ ≈ 25–62 months), indicating their greater stability in dried BP. In particular, aspartic acid (t_1/2_ = 62.06 months) and histidine (t_1/2_ = 50.29 months) showed exceptionally high stability (Table 4, Figure 8).

In this study, Q_10_ values were calculated to quantitatively evaluate the effect of temperature on amino acid degradation rates under different storage conditions: from −80 °C to −20 °C, from −20 °C to room temperature (RT), and across the entire temperature range from −80 °C to room temperature (RT) (Table 5). For most amino acids, Q_10_(−80 → −20) values were close to unity (0.95–1.05), indicating that degradation rates changed only marginally between −80 °C and −20 °C. This suggests that ultra-low temperature storage at −80 °C did not provide a substantial additional stabilizing effect compared with −20 °C for the majority of amino acids. For certain compounds, such as methionine and arginine, Q_10_ values were below 1, indicating slightly higher degradation rates at −80 °C, possibly due to matrix-specific effects or oxidative processes. In contrast, Q_10_(−20 → RT) values were greater than 1 for most amino acids, frequently ranging from 1.1 to 1.3 and, in some cases, exceeding these values. This clearly demonstrates that the transition from frozen storage to room temperature markedly accelerated amino acid degradation. A particularly pronounced temperature effect was observed for tyrosine (Q_10_ = 1.801) and methionine (Q_10_ = 1.288), confirming the high sensitivity of these amino acids to oxidative and non-enzymatic degradation processes at elevated temperatures.

Overall, Q_1__0_(−80 → RT) values for most amino acids ranged between approximately 1.0 and 1.1, indicating a moderate but consistent increase in degradation rates with rising temperature across the investigated range. These findings confirm that the most substantial loss of amino acid stability occurs during the transition from frozen storage to room temperature rather than between different low-temperature storage regimes.

Overall, the results demonstrate that storage temperature and preservation method significantly influence the stability of free amino acids in bee pollen. Sulfur-containing amino acids, particularly cysteine and methionine, showed the highest degradation rates and the shortest half-lives under all storage conditions, indicating pronounced instability. In contrast, alanine, glutamic acid, proline, serine, and phenylalanine exhibited low degradation rate constants and long half-lives, reflecting high stability. Frozen storage at −20 °C and −80 °C effectively reduced amino acid degradation compared to room-temperature storage; however, no consistent advantage of −80 °C over −20 °C was observed for all amino acids. The first-order kinetic model adequately described amino acid degradation behavior across all storage conditions.

## 4. Discussion

Bee pollen is a complex biochemical matrix whose composition is influenced by botanical origin, geographic region, environmental conditions, and even the specific bee colony. Studies have shown that the chemical composition of BP, particularly its amino acid profile, can vary among colonies located within the same area and exposed to similar floral and environmental conditions [30,52,53,54,55]. For example, aloe (*Aloe* spp.) pollen, collected by worker bees, is characterized by a high protein content of approximately 51%, whereas sunflower (*Helianthus annuus* L.) pollen contains a significantly lower amount (around 26%) [56]. Similarly, *Echium plantagineum* L. pollen has a relatively high protein content (37.4%) [57], while buckwheat (*Fagopyrum esculentum* Moench) pollen contains less protein (11.4%) but exhibits a more diverse amino acid composition, particularly rich in essential amino acids, thereby enhancing its nutritional value [57]. These findings indicate that the nutritional quality of BP depends not only on total protein concentration but also on the qualitative composition of amino acids and their biological availability [57]. Such variability can be attributed to species-specific physiological characteristics, enzymatic activity, and the botanical origin of the collected pollen. In this context, it should be emphasized that the findings of the present study primarily reflect the behavior of free amino acids in bee-collected pollen dominated by *Brassica napus* and *Salix* spp. Given the well-documented influence of botanical origin on pollen chemical composition, extrapolation of these results to bee pollen with substantially different botanical profiles should therefore be made with caution.

Using ultra-high performance liquid chromatography with electrospray ionisation mass spectrometry (UHPLC-ESI-MS/MS), 17 free amino acids were identified in BP samples collected in Lithuania. These comprised nine essential amino acids (EAAs)—Arg, His, Ile, Leu, Lys, Met, Phe, Thr, and Val—which cannot be synthesized by the human body and must therefore be obtained through the diet, and eight non-essential amino acids (NEAAs)—Ala, Asp, Cys, Glu, Gly, Pro, Ser, and Tyr. Similar results have been reported in other studies analyzing the amino acid composition of BP samples. Hsu et al., identified 18 amino acids in BP collected in Taiwan, comprising 10 EAAs and 8 NEAAs [58]. Likewise, Oroian et al. reported 16 amino acids in BP samples from Romania, including 8 EAAs and 8 NEAAs [59]. Nevertheless, some studies have demonstrated that BP samples may contain a broader spectrum of amino acids. For example, Rzetecka et al., reported the identification of 32 amino acids in BP samples collected in Poland [60]. These findings suggest that the amino acid profile of BP can vary considerably depending on geographical origin, botanical source, and the sensitivity and specificity of analytical methods used.

The total content of free amino acids in BP samples collected in Lithuania ranged from 7352.02 ± 367.60 µg/g to 8073.97 ± 403.70 µg/g, which is comparable to the concentrations reported in Romanian BP samples (11,310–45,990 µg/g) [59]. However, these levels are approximately 3 to 4.7 times lower than those determined in BP samples from Colombia (25,300 µg/g), Italy (29,400 µg/g), and Spain (30,800 µg/g) [43].

In the BP samples collected in Lithuania, three predominant free amino acids were identified: arginine (1145.01 ± 57.25 µg/g—1216.60 ± 60.83 µg/g), proline (1026.65 ± 51.33 µg/g—1176.03 ± 58.80 µg/g), and aspartic acid (903.76 ± 45.19 µg/g—965.79 ± 48.29 µg/g). These findings are consistent with previous studies conducted in Türkiye, where the major amino acids detected in BP samples were proline (8384.22–16,670.79 µg/g) and aspartic acid (3669.36–5260.56 µg/g) [32]. In the Lithuania collected BP samples the predominant amino acid was the essential amino acid arginine, accounting for 14.5–15.8% of the total amino acid content. Arginine was identified as the predominant amino acid in BP samples from *Fagopyrum esculentum* Moench, *Vicia faba* L., *Helianthus annuus* L., and *Zea mays* L. species [17,30,57,58,61,62]. In BP samples collected from different regions of Türkiye, the arginine content (186.70–744.51 µg/g) was found to be 1.6–6.1 times lower than that determined in the Lithuanian samples. Our findings contradict the data reported in scientific literature, which indicate that the predominant essential amino acid in BP samples is histidine [56]. The proportion of essential amino acids to total amino acids (EAA/TAA) in Lithuanian BP samples (47–48%) was higher than that reported for rapeseed, maize, and sunflower pollens (39–45%, 38–41%, and 36–43%, respectively) [63], indicating a relatively richer essential amino acid composition in Lithuanian bee pollen.

Proline was the second most abundant amino acid in the BP samples, following arginine, accounting for 14.0–14.6% of the total amino acid content. Several scientific studies have reported that proline is the predominant amino acid in BP samples [32,60,63,64,65]. The proline content determined in BP samples collected in Lithuania was similar to that reported for BP samples from Spain (970–2870 µg/g) [65], but was 4.1–7.7 times lower than in Polish BP samples (4220–9080 µg/g) [60] and 4.8–18.9 times lower than in BP samples from Türkiye (4937–22,212 µg/g) [32]. When evaluating the amino acid profile of BP samples collected in Lithuania, the lowest concentration was observed for cysteine, ranging from 10.19 ± 0.50 µg/g to 14.18 ± 0.71 µg/g. According to Jeannerod et al., the analysis of pollen from various plant species revealed the presence of all essential amino acids and nearly all other amino acids, except for cysteine [66].

The physicochemical stability of bee-collected pollen is strongly influenced by processing techniques [7,40,42,67,68,69,70] and storage conditions [71,72,73], which are essential to prevent microbial growth and biochemical degradation [35,36]. Fresh pollen contains up to 30% moisture [74], requiring prompt drying to ensure stability. However, conventional hot air drying may induce Maillard reactions, oxidation, and nutrient loss, particularly affecting amino acids, lipids, and antioxidants. To mitigate these effects, low-temperature drying techniques, such as freeze-drying, have been developed to better preserve proteins, vitamins, and bioactive compounds. Proper drying and storage are therefore crucial for maintaining the nutritional quality and biochemical integrity of BP during long-term preservation.

In both frozen and dried BP samples, the total free amino acid content (TAAC) decreased statistically significantly after nine months of storage under the respective conditions (*p* < 0.05). After nine months, a 1.1-fold decrease was observed in the dried samples, while the samples stored at −20 °C and −80 °C exhibited 1.2-fold and 1.3-fold decreases, respectively (*p* < 0.05). Prolonged storage further intensified these changes: after 15 months, the TAAC decreased 1.5-fold in samples stored at −20 °C and 1.7-fold in those stored at −80 °C (*p* < 0.05). A similar trend was observed in the dried BP samples, where a 1.6-fold reduction was detected after 15 months of storage. These findings demonstrate that extended storage duration and lower storage temperatures lead to a gradual yet statistically significant decline in the TAAC of BP samples.

After 15 months of storage, clear differences in the stability of individual free amino acids were observed depending on storage conditions and temperature. Frozen BP samples exhibited markedly lower degradation compared to dried samples stored at room temperature, a trend consistently supported by both concentration changes and kinetic parameters derived from the first-order degradation model. Among frozen samples, Ala, Glu, and Pro showed the smallest reductions in concentration, with decreases not exceeding 15–19% at −20 °C and −80 °C. These observations are quantitatively reflected by their low degradation rate constants and long half-lives, confirming their high stability under frozen storage conditions.

The application of first-order kinetic modeling enabled a detailed, compound-specific evaluation of amino acid stability. Amino acids characterized by low degradation rate constants (*k* ≈ 0.010–0.017 month^−1^) exhibited half-lives exceeding 40 months, indicating limited susceptibility to degradation over the investigated storage period. In contrast, sulfur-containing amino acids—particularly Cys and Met—displayed the highest degradation rate constants and the shortest half-lives across all storage conditions. Even under frozen storage, Cys degradation was rapid, reflecting its intrinsic chemical instability associated with the presence of a reactive thiol group. These findings demonstrate that freezing substantially slows, but does not fully prevent, degradation processes for chemically labile amino acids.

The observed stability patterns are consistent with known structure–reactivity relationships. Small or chemically inert amino acids, such as alanine, exhibit reduced susceptibility to oxidation and secondary reactions due to their simple molecular structure, whereas amino acids containing sulfur or highly reactive functional groups are more prone to oxidative and degradative pathways [40,75]. The stability of Pro under frozen conditions may further be attributed to its cyclic structure, which confers resistance to both oxidative and Maillard-type reactions. Comparable trends were reported by Dias et al. [76], who observed moderate reductions in proline content during short-term storage of frozen and dried BP samples. However, the longer storage duration in the present study reveals that compound-specific differences become more pronounced over extended periods, underscoring the importance of long-term investigations.

In dried BP samples stored at room temperature, degradation patterns differed substantially. Although overall losses were greater, certain amino acids—such as Asp, His, and Val—retained comparatively higher stability, as indicated by their lower degradation rate constants and longer half-lives. This behavior can be linked to structural features, including increased polarity (aspartic acid), buffering and metal-chelating capacity (histidine), or chemically inert aliphatic side chains (valine), which may mitigate degradation under non-frozen conditions [38,75]. Nevertheless, kinetic parameters clearly indicate that room-temperature storage accelerates degradation for most amino acids, particularly those prone to oxidation or secondary reactions.

Temperature dependence of amino acid degradation was further elucidated using Q_10_ values, allowing quantitative comparison of degradation sensitivity across temperature intervals. For most amino acids, Q_10_ values close to unity were observed for the −80 °C to −20 °C transition, indicating that lowering the temperature below −20 °C provided only marginal additional stabilization. In contrast, substantially higher Q_10_ values were obtained for the −20 °C to room temperature transition, especially for Tyr, Met, and Cys, highlighting the pronounced acceleration of degradation processes under non-frozen conditions. These results demonstrate that the primary protective effect is achieved by freezing itself, whereas ultra-low temperatures offer compound-specific rather than universal improvements in stability.

Overall, the combined application of first-order kinetic modeling, half-life estimation, and Q_10_ analysis provides a robust, data-driven framework for interpreting amino acid stability in BP. Rather than relying on qualitative trends alone, this integrated approach enables direct comparison of degradation behavior across amino acids and storage conditions, highlighting the dominant role of temperature and intrinsic molecular properties in determining long-term stability. The findings emphasize that while frozen storage effectively preserves the overall amino acid profile, chemically labile amino acids—particularly sulfur-containing residues—remain vulnerable even under ultra-low temperatures.

## 5. Conclusions

Seventeen free amino acids, including all nine essential amino acids, were identified in Lithuanian bee pollen, with essential amino acids accounting for 47–48% of the total amino acid content. Arginine, proline, and aspartic acid were the dominant free amino acids. Long-term storage significantly affected amino acid stability, with total amino acid content decreasing by 1.5–1.7-fold after 15 months. Frozen storage at −20 °C and −80 °C was markedly more effective than drying at preserving nutritionally important amino acids, particularly alanine, glutamic acid, and proline, whereas storage of dried bee pollen at room temperature led to pronounced degradation. Sulfur-containing amino acids, such as cysteine and methionine, were the most unstable and may serve as sensitive indicators of oxidative deterioration during storage. From a practical perspective, these findings indicate that frozen storage, even at −20 °C, is sufficient to substantially preserve amino acid quality in BP, while room-temperature storage should be carefully controlled to minimize nutritional losses. The results are directly applicable to the food and nutraceutical industries for optimizing storage conditions and ensuring the quality of bee pollen products.

## Figures and Tables

**Table 1:** Mass spectrometer parameters for amino acid analysis.

No.	Compound	Precursor Ion (m/z)	Product Ion (m/z)	Cone Voltage, V	Collision Energy, eV
1	Alanine	90	44	16	10
2	Arginine	175	70	16	14
3	Aspartic acid	134	88	14	10
4	Cysteine	122	76	14	17
5	Glutamic acid	148	84	12	14
6	Glycine	76	30	12	6
7	Histidine	156	110	20	16
8	Isoleucine	132	86	16	10
9	Leucine	132	86	16	10
10	Lysine	147	84	14	14
11	Methionine	150	104	14	10
12	Phenylalanine	166	120	18	14
13	Proline	116	70	20	10
14	Serine	106	60	14	8
15	Threonine	120	74	38	20
16	Tyrosine	182	136	16	16
17	Valine	118	72	12	10

**Table 2:** Parameters of the first-order degradation model for free amino acids in BP samples stored at −20 °C.

No.	Amino Acid	*C*_0_ (µg/g)	*k* (Month^−1^)	*R* ^2^	*t*_1/2_ (Months)
1	Ala	519.5	0.0104	0.9282	66.36
2	Arg	1145.0	0.0339	0.9216	20.48
3	Asp	965.8	0.0209	0.9857	33.22
4	Cys	14.2	0.4488	0.9444	1.54
5	Glu	740.6	0.0134	0.9658	51.57
6	Gly	297.2	0.0371	0.9256	18.67
7	His	599.3	0.0343	0.6817	20.23
8	Ile	147.9	0.0870	0.9541	7.97
9	Leu	470.3	0.0924	0.8651	7.50
10	Lys	256.3	0.0408	0.9099	17.00
11	Met	191.7	0.0502	0.9315	13.81
12	Phe	307.4	0.0170	0.9629	40.81
13	Pro	1133.9	0.0133	0.8776	51.93
14	Ser	397.7	0.0165	0.9487	42.08
15	Thr	187.9	0.0511	0.9941	13.56
16	Tyr	150.4	0.0303	0.9624	22.87
17	Val	354.2	0.0395	0.7576	17.55

**Table 3:** Parameters of the first-order degradation model for free amino acids in BP samples stored at −80 °C.

No.	Amino Acid	*C*_0_ (µg/g)	*k* (Month^−1^)	*R* ^2^	*t*_1/2_ (Months)
1	Ala	518.5	0.0122	0.9594	56.86
2	Arg	1216.6	0.0837	0.9200	8.29
3	Asp	930.4	0.0232	0.9629	29.88
4	Cys	13.7	0.6025	0.9462	1.15
5	Glu	759.4	0.0104	0.9793	66.51
6	Gly	319.4	0.0308	0.8150	22.54
7	His	627.0	0.0478	0.8502	14.50
8	Ile	160.3	0.1022	0.9864	6.79
9	Leu	439.9	0.0942	0.8545	7.36
10	Lys	261.4	0.0296	0.9310	23.39
11	Met	199.1	0.1756	0.8132	3.95
12	Phe	316.7	0.0146	0.9622	47.61
13	Pro	1176.0	0.0149	0.8153	46.65
14	Ser	411.2	0.0173	0.9440	40.18
15	Thr	194.5	0.0343	0.8976	20.20
16	Tyr	170.5	0.0294	0.9390	23.55
17	Val	359.4	0.0487	0.7765	14.22

**Table 4:** Parameters of the first-order degradation model for free amino acids in dried BP samples.

No.	Amino Acid	*C*_0_ (µg/g)	*k* (Month^−1^)	*R* ^2^	*t*_1/2_ (Months)
1	Ala	498.9	0.0270	0.8564	25.66
2	Arg	1164.5	0.0308	0.8890	22.5
3	Asp	903.8	0.0112	0.8527	62.06
4	Cys	10.2	0.9024	1.0000	0.77
5	Glu	696.1	0.0253	0.9979	27.35
6	Gly	281.1	0.0849	0.8699	8.16
7	His	568.7	0.0138	0.7486	50.29
8	Ile	129.1	0.0589	0.7577	11.78
9	Leu	420.0	0.0238	0.9348	29.18
10	Lys	244.6	0.0553	0.8916	12.53
11	Met	188.5	0.1380	0.9083	5.02
12	Phe	280.2	0.0285	0.9928	24.28
13	Pro	1026.7	0.0246	0.8595	28.14
14	Ser	354.8	0.0340	0.9134	20.36
15	Thr	172.1	0.0819	0.8892	8.46
16	Tyr	62.5	0.3189	0.9758	2.17
17	Val	350.4	0.0187	0.7457	36.99

**Table 5:** Temperature coefficients (Q_1__0_) for amino acid degradation in bee pollen stored at −80 °C, −20 °C, and room temperature.

No.	Amino Acid	Q_1__0_ (−80 → −20)	Q_1__0_ (−20 → RT)	Q_1__0_ (−80 → RT)
1	Ala	0.975	1.268	1.083
2	Arg	0.860	0.977	0.905
3	Asp	0.982	0.855	0.930
4	Cys	0.952	1.191	1.041
5	Glu	1.043	1.172	1.093
6	Gly	1.032	1.230	1.107
7	His	0.946	0.796	0.883
8	Ile	0.974	0.907	0.946
9	Leu	0.997	0.712	0.871
10	Lys	1.055	1.079	1.064
11	Met	0.812	1.288	0.976
12	Phe	1.026	1.139	1.070
13	Pro	0.982	1.166	1.052
14	Ser	0.992	1.199	1.070
15	Thr	1.069	1.125	1.091
16	Tyr	1.005	1.801	1.269
17	Val	0.966	0.830	0.909

RT—room temperature.

## Data Availability

The data are contained within the article.

## Supplementary Materials

The following supporting information can be downloaded at: https://www.mdpi.com/article/10.3390/foods15020207/s1, Table S1. Summary of published studies investigating amino acid–related changes in bee pollen under different processing and storage conditions.
