# Supplementary material for: Changes in the Amino Acid Composition of Bee-Collected Pollen During 15 Months of Storage in Fresh-Frozen and Dried Forms
Source: Foods. 2026 Jan 7;15(2):207. doi: 10.3390/foods15020207 (PMC12840115; doi:10.3390/foods15020207)
Supplement: Supplementary file 1 [file foods-15-00207-s001.zip › foods-4054678-supplementary.pdf]

Table S1. Summary of published studies investigating amino acid–related changes in bee pollen under different processing and storage conditions.

Reference	Matrix	Processing / storage conditions	Storage duration	Amino acid–related parameters evaluated	Main limitations
Castagna A. et al., 2020 [23].	Bee pollen	Hot air drying; freeze-drying	≤6 months	Amino acid profile	Short storage duration; no frozen storage
Gardana C. et al., 2018 [43].	Bee pollen	Different botanical origins	Not storage-focused	Amino acid composition	No processing or storage evaluation
Anjos O. et al., 2023 [44].	Bee pollen	Drying; high-pressure processing	≤2 months	Total amino acids	No individual FAA analysis
Canale A. et al., 2016 [45].	Bee pollen	Microwave-assisted drying	Immediate / short-term	Total amino acids	No storage study; no FAA analysis
Rzepecka-Stojko A. et al., 2022 [46].	Bee pollen	Drying and room-temperature storage	≤3 months	Selected amino acids	Short duration; limited FAA scope
Denisow B. et al., 2023 [47].	Bee pollen	Conventional drying	≤6 months	Protein-related indices	No frozen storage; no kinetics
Present study	Bee pollen	−20 °C and −80 °C freezing; low-temperature drying	15 months	Free amino acids; EAA/NEAA; degradation kinetics	—

FAA – free amino acids; EAA – essential amino acids; NEAA – non-essential amino acids
